# DNA replication and replication stress response in the context of nuclear architecture

**DOI:** 10.1007/s00412-023-00813-7

**Published:** 2023-12-06

**Authors:** Daniel González-Acosta, Massimo Lopes

**Affiliations:** Institute of Molecular Cancer Research, University of Zurich, Zurich, Switzerland

**Keywords:** DNA replication, Replication stress, Nuclear dynamics, Genome organisation, Nuclear architecture

## Abstract

The DNA replication process needs to be coordinated with other DNA metabolism transactions and must eventually extend to the full genome, regardless of chromatin status, gene expression, secondary structures and DNA lesions. Completeness and accuracy of DNA replication are crucial to maintain genome integrity, limiting transformation in normal cells and offering targeting opportunities for proliferating cancer cells. DNA replication is thus tightly coordinated with chromatin dynamics and 3D genome architecture, and we are only beginning to understand the underlying molecular mechanisms. While much has recently been discovered on how DNA replication initiation is organised and modulated in different genomic regions and nuclear territories—the so-called “DNA replication program”—we know much less on how the elongation of ongoing replication forks and particularly the response to replication obstacles is affected by the local nuclear organisation. Also, it is still elusive how specific components of nuclear architecture participate in the replication stress response. Here, we review known mechanisms and factors orchestrating replication initiation, and replication fork progression upon stress, focusing on recent evidence linking genome organisation and nuclear architecture with the cellular responses to replication interference, and highlighting open questions and future challenges to explore this exciting new avenue of research.

## Introduction

Replication of the mammalian genome is a highly complex process, occurring with remarkable accuracy during the S phase of the cell cycle. Due to the large size of eukaryotic genomes, DNA replication starts at multiple sites, known as “replication origins” (Aladjem and Redon 2017). Initiation of DNA synthesis is strictly regulated in time and space, following a replication timing program that determines the regions initiating at a given time through the S phase. Units of replication timing (RT) correspond to structural units of genome folding called topologically associating domains (TADs)—spanning from 100 kb to few megabases—which constitute regions of high internal frequency of interaction, compared to interactions established with genomic locations outside the TAD. However, determining the precise location of origins along the genome and specifically their organisation inside TADs is a challenging endeavour and a matter of intense research (Vouzas and Gilbert 2021). Due to the role of 3D genome folding and interactions, it is not surprising that factors determining chromatin architecture and nuclear dynamics have been implicated in the DNA replication process.

Much less is known about the impact that structural factors have on replication forks facing obstacles. Stressed replication forks can deal with impediments in multiple ways, frequently involving active fork slowing and remodelling into four-way junctions, or recruiting factors that sustain active fork progression upon stress. How the cells balance different mechanisms of replication stress tolerance is poorly understood (Berti et al. 2020). However, the recurrent observation that different DNA metabolic processes such as DNA replication and recombination are regulated in the frame of structural units—rather than as individual events—implicates genome architectural factors as crucial components of the global replication stress response (Mamberti and Cardoso 2020).

In this review, we discuss how DNA replication is prepared and started, and how cells cope with replication interference, in the frame of nuclear architecture. Recapitulating basic mechanisms of DNA replication control, we integrate the latest insights on how different factors governing chromosome organisation and nuclear structure help regulating these crucial processes. While the role of histone modifications in replication fork integrity has been extensively reviewed elsewhere (Wootton and Soutoglou 2021), we mention here those that are particularly relevant in modulating replication timing and stress tolerance in the context of nuclear architecture. We comment on possible future directions and open questions in this new and exciting avenue of research.

## Preparing replication origins for S phase

### Nucleosomal density and replication initiation

During late mitosis and along G1 phase of the cell cycle, nascent and parental MCM2-7 complexes—the core component of the replicative helicase—are loaded on chromatin by the action of the two helicase-loading factors CDC6 and CDT1 (Ekundayo and Bleichert 2019; Sedlackova et al. 2020). Both factors cooperate with the origin recognition complex (ORC1-6) to load two MCM double hexamers (MCM DHs) in a head-to-head conformation forming the pre-replication complex (pre-RC). The pre-RC marks licensed origins, i.e. those potentially able to fire in the following S phase (Costa and Diffley 2022). When loaded, ring-shaped MCM complexes encircle dsDNA and transition to ssDNA upon activation of the replicative helicase and initiation of DNA synthesis (Costa and Diffley 2022). Surprisingly, in some cancer cell lines, ORC subunits 1 and 2 seem to be dispensable for cell survival, as they strongly rely on CDC6 to load a reduced—albeit sufficient—amount of MCM to support DNA replication (Shibata et al. 2016).

**Fig. 1:**
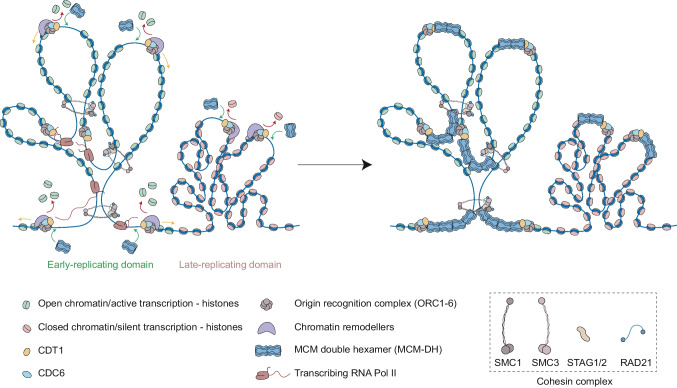
Replication origin licensing in early and late-replicating regions. Early and late-replicating domains are enriched in open and closed chromatin histone marks respectively. Thus, the former is a more decondensed, transcriptionally active chromatin environment compared to the latter. ORC complex is bound to chromatin throughout the cell cycle in nucleosome-free regions, and together with CDT1 promotes the action of chromatin remodellers, which promote unloading (red arrow) or repositioning (orange arrow) of nucleosomes in G1 to facilitate pre-RC formation. Several MCM DH can be loaded (green arrow) at every ORC position, and the action of cohesin-mediated loop extrusion could help to retain them at origin positions, limiting their spreading. The accessibility of early-replicating regions and their higher transcriptional activity might favour a higher enrichment of pre-RC components in these regions compared to late-replicating ones

ORC remains bound to chromatin during the whole cell cycle (Méndez and Stillman 2000), with the exception of ORC1 which is bound in G1 and is degraded throughout the S phase (Méndez et al. 2002), determining where pre-RC will be formed. Profiling of ORC1 and ORC2 binding positions in human cells revealed that ORC binds preferably to DNAse I hypersensitive regions, enriched in acetylated H3 (H3K27 and H3K9) and mono-, di- and tri-methylated H3K4. Although a correlation of ORC binding with transcription starting sites (TSS) was found, no direct link was found between them (Dellino et al. 2013; Miotto et al. 2016). A direct implication of ORC5 in chromatin de-condensation through GCN5 has been proposed (Giri et al. 2015b), but ORC is most likely to take advantage of an already open chromatin environment to bind these regions. Moreover, the histone variant H2A.Z was shown to cooperate with SUV420H1 to di-methylate H4K20 and promote origin licensing at early-replicating origins by recruiting ORC1 through a direct interaction with H4K20me2 (Kuo et al. 2012; Long et al. 2020). Accordingly, a greater amount of ORC positions has been found in early-replicating, highly transcribed regions compared to late-replicating, silent chromatin, suggesting a possible correlation with RT patterns (Dellino et al. 2013; Gindin et al. 2014; Miotto et al. 2016) (Fig. 1).

Given the small width of its ring channel, the MCM complex cannot accommodate nucleosomes in the interior; hence, MCM DHs must be loaded on nucleosome-free DNA region (Hyrien 2016). ORC binding to open chromatin regions would facilitate MCM loading, but further remodelling of those genomic positions appears to be needed to achieve complete and successful pre-RC loading. Different histone-modifying enzymes or chromatin remodelling complexes have been involved in regulating replication origin licensing and firing.

SAF-A/HNRNPU, a protein involved in chromatin de-condensation, was shown to contribute to ORC and MCM loading onto chromatin, likely by generating a suitable open chromatin environment (Connolly et al. 2022). Also, human acetylase binding to ORC1 (HBO1) enzyme, which specifically acetylates histone H4, binds to at least a subset of replication origins in the G1 phase, possibly located near promoters, due to direct interaction with CDT1. Through its acetylase activity, HBO1 was shown to facilitate pre-RC formation at replication origins (Miotto and Struhl 2010) and to promote their activation via its interaction with BRPF3 (Feng et al. 2016). This might be achieved in cooperation with chromatin remodellers such as CDT1-interacting proteins SNF2H, WSTF and GRWD1, which prefer binding acetylated histones (Sugimoto et al. 2008, 2011, 2015). Ultimately, this would facilitate the loading of MCM DHs on genomic DNA (Fig. 1).

Nucleosome remodelling might be particularly important at late-replicating regions, mainly composed of heterochromatin with a less open chromatin environment. This feature of heterochromatin likely limits the loading of ORC and MCM DHs, hampering firing throughout the S phase, and leading to a later replication timing. In line with this, ORC-associated (ORCA) protein, which interacts simultaneously with ORC and heterochromatin marks such as H3K20me3, prevents heterochromatin compaction and promotes the stabilisation of histone methyltransferase complexes that favour chromatin reorganisation at late-replicating regions. This in turn facilitates the loading of pre-RC components such as MCMs at those positions (Shen et al. 2010, 2012; Giri et al. 2015a; Wang et al. 2016; Mei et al. 2022; Sahu et al. 2023). However, fine tuning of ORC and MCM DH loading appears to be required to maintain proper genome stability, since excessive decompaction by removing H4K20 methyltransferase SET8/PR-SET7 leads to pre-RC overloading and DNA damage in the following S phase (Shoaib et al. 2018). Prolonged depletion of SET8/PR-SET7 also has a negative impact on pre-RC loading, emphasising the critical role of fine-tuned chromatin organisation in the loading of origin licensing factors (Tardat et al. 2007, 2010; Beck et al. 2012).

The amount of MCM proteins loaded at the end of G1 greatly exceeds the levels of ORC on chromatin, indicating that more than one MCM DH could be loaded at every ORC position (Hyrien 2016). In line with ORC-binding pattern and chromatin openness, MCMs are also found to be more enriched at early- vs late-replicating initiation zones (Kirstein et al. 2021). In yeast, higher enrichment of MCM complexes at early-replicating regions has a direct impact on the replication timing program (Das et al. 2015). This correlation per se does not explain RT patterns in human cells (Kirstein et al. 2021). However, a recent study revealed that severe depletion of MCM complex abolishes RT patterns, suggesting that differential enrichment of MCMs could have at least an indirect role on RT (Peycheva et al. 2022). It is possible that the open chromatin environment bound by ORC promotes the activation of the MCM DHs loaded at those regions, leading to earlier replication.

### The role of chromatin architecture in origin determination

Beside nucleosome remodellers and histone modifiers, proteins with architectural roles have been shown to interact with pre-RCs likely regulating their positioning in G1 and their activation in the following S phase. Whether these roles are directly or indirectly driven by their function in genome organisation is difficult to discern, but recent studies have shed some light on this question.

Initial studies identified a role for the cohesin complex in the regulation of origin firing (Guillou et al. 2010). Cohesin is a ring-shaped multiprotein complex involved in genome architecture due to its ability to form DNA loops, bringing distant DNA elements close together. This so-called “loop extrusion” function of cohesin is dependent on NIPBL/Scc2, frequently mutated in Cornelia de Lange Syndrome (CdLS) patients (Davidson and Peters 2021). Cohesin has been shown to interact with the MCM helicase (Guillou et al. 2010; Zheng et al. 2018), which is impaired by prolonged NIBPL depletions (Zheng et al. 2018). Recent studies using Hi-C and single-molecule imaging experiments show that the MCM complex is a physical barrier for loop extruding cohesin (Dequeker et al. 2022), as previously shown for converging CTCF binding sites (Gassler et al. 2017; Nora et al. 2017; Wutz et al. 2017) and transcription (Jeppsson et al. 2022; Banigan et al. 2023). It seems unlikely that the MCM complex is a key player in genome organisation by establishing cohesin positions, as MCM depletion affects loop formation but only mildly TAD conformation and insulation (Dequeker et al. 2022; Peycheva et al. 2022). Conversely, these data rather suggest that cohesin could help establishing the localisation and concentration of MCM DHs (Fig. 1). In line with this scenario, cohesin subunit RAD21 and CTCF are reportedly interacting with SAF-A/HNRNPU, which could help establishing a permissive environment to locate MCM DHs (Fan et al. 2018).

The architectural factor RIF1 is one of those with the strongest described impact on the replication program. Besides its established roles in telomere maintenance and DSB repair, RIF1 has been involved in genome folding and replication timing (Blasiak et al. 2021; Richards et al. 2022). Together with protein phosphatase 1 (PPT1), RIF1 protects ORC1 from degradation in G1, promoting its chromatin association and MCM complex loading (Hiraga et al. 2017). Since RIF1 was found to bind a subset of wide late-replicating regions (Foti et al. 2016), it is conceivable that it regulates pre-RC component loading in those regions. However, the association of ORC1 and MCM proteins to chromatin and the rate of DNA synthesis are greatly reduced upon RIF1 depletion, suggesting a global effect on origin licensing or firing, rather than a specific effect on RIF1-bound regions (Hiraga et al. 2017). However, prolonged siRNA-mediated depletion used in this study may also lead to substantial epigenomic changes, indirectly affecting pre-RC formation. Alternatively, RIF1 may globally affect origin firing by a mechanism independent from its chromatin association or its binding positions may be reorganised upon entry into S phase.

## Initiating, extending and completing DNA synthesis

The onset of the S phase is marked by the increase in cyclin-dependent kinase (CDK) and DBF4-dependent kinase (DDK) CDC7 activities, which prevent the assembly of new pre-RCs and promote the conversion of previously formed pre-RCs into pre-initiation complexes (pre-ICs), by phosphorylating different pre-RC components. The pre-IC complex is formed when CDC45 and GINS tetramer (SLD5, PFS1, PFS2 and PFS3) are assembled with the MCM complex forming the “CMG replicative helicase”. The loading of additional factors—including MCM10, RPA and polymerase ε—promotes the activation of the CMG helicase and prepares the “replisome” for DNA synthesis (Costa and Diffley 2022). Binding of the polymerase α -primase complex finally initiates DNA synthesis bidirectionally from each replication origin, which is extended by POLε in the continuous “leading” strand and POLδ in the discontinuous “lagging” strand (Burgers and Kunkel 2016).

Mapping exactly where the initiation of DNA synthesis takes place genome-wide proved challenging, with surprisingly low overlap among initiation sites identified by different approaches (Ganier et al. 2019; Hu and Stillman 2023). Mainly, six different techniques, leveraging on distinctive features of replication forks to map mammalian initiation sites genome-wide, have been used:

1. Short nascent strand sequencing (SNS-seq): isolation of RNA-protected DNA primers synthesised at the leading strand (Gómez and Brockdorff 2004; Sequeira-Mendes et al. 2009; Cayrou et al. 2011; Martin et al. 2011; Besnard et al. 2012; Smith et al. 2016; Akerman et al. 2020; Jodkowska et al. 2022)
2. Replication bubble sequencing (Bubble-seq): After digestion with restriction enzymes, replication bubbles are trapped in an agarose gel, isolated and sequenced (Mesner et al. 2013)
3. Initiation sequencing (Ini-seq): isolation of BrdUTP-labelled regions after a short pulse in the early S phase (Langley et al. 2016; Guilbaud et al. 2022)
4. Okazaki fragment sequencing (OK-seq): isolation of Okazaki fragments to identify the directionality of replication forks on the genome. Changes in directionality indicate the presence of an initiation site (Petryk et al. 2016)
5. EdU sequencing (EdU-seq): Biotin linkage to EdU by click chemistry allows immunoprecipitation of short EdU-labelled region by streptavidin capture (Macheret and Halazonetis 2018)
6. Optical replication mapping (ORM): after a short pulse of early S phase cells with a fluorescent nucleotide analog, DNA fibres containing replication tracks are analysed by optical mapping technologies (Wang et al. 2021).

**Fig. 2:**
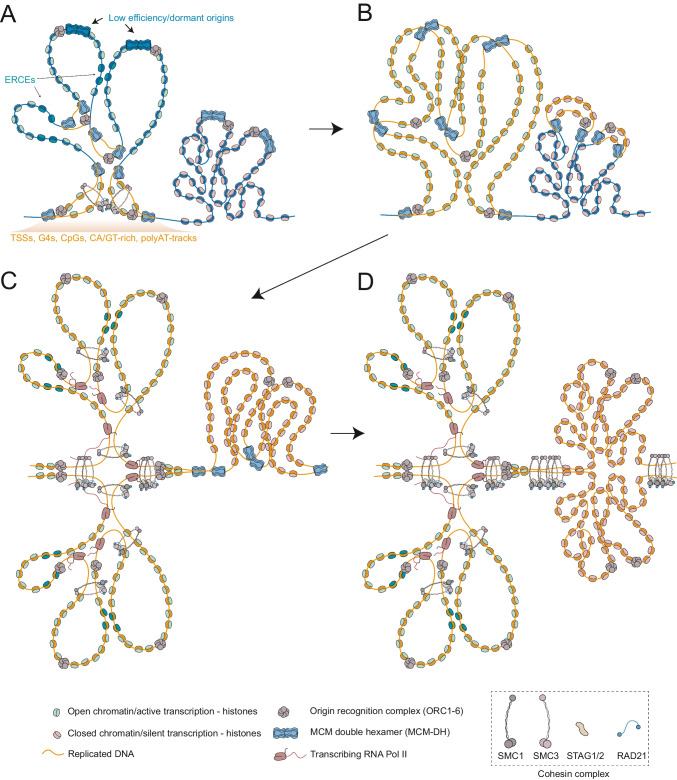
Initiation and progression of DNA synthesis in early and late-replicating domains. **A** Initiation sites are associated with the presence of TSSs, G-quadruplexes, CpG islands, CA/GT-rich sequences or polyAT-tracks among others. However, none of them is per se a mandatory indicator of the presence of a replication origin. Genetic elements such as ERCEs are strong regulators of the RT of a region. Efficient origins locate at TAD borders or tend to locate at the base of replication domains. Low-efficiency origins fire in a small percentage of cells in the population and probably constitute back-up, dormant origins in case of problematic synthesis in neighbouring replication forks. **B** Upon replication completion of early-replicating regions, those will undergo reorganisation while late-replicating regions initiate DNA synthesis. This temporal separation likely favours the re-establishment of open chromatin marks, intra-TAD interactions and transcription re-initiation at early replication domains (as shown in **C**). Moreover, it enables proper use of limited replication resources along the S phase. **D** At the end of S phase, both sister chromatids have completely re-established intra-TAD *cis* contacts and cohesive cohesin is placed at TAD boundaries

However, from these different techniques, it has become clear that most initiation regions contain common genetic and epigenetic features (Ganier et al. 2019; Hu and Stillman 2023). In agreement with ORC-binding sites, they are mainly located at regions associated with active transcription and promoters, open chromatin marks, DNase I hypersensitivity, commonly rich in G/C nucleotide sequences. The presence of CpG islands and G4 structures correlates with active replication origins but is not a strict requirement (Ganier et al. 2019). Moreover, in mouse cells, replication initiation sites have been linked to the presence of CA/GT-rich (Cayrou et al. 2015) and poly-A/T tracks (Tubbs et al. 2018) (Fig. 2A).

Among those techniques, only SNS-seq and OK-seq are able to map initiation sites along the whole S phase. SNS-seq is the technique with highest resolution (< 2 Kb), identifying between 40,000 to 200,000 positions genome-wide in mammalian cell lines, whereas the low resolution of OK-seq (34–150 Kb) allows to identify initiation domains (Ganier et al. 2019). From studies using SNS-seq or Ini-seq, a set of “common/constitutive” high-efficiency initiation sites, i.e. those ones used in most of the cells in the population, have been described. Additionally, a recent study using SNS-seq in several human cell lines described the presence of “core” origins, which constitute highly efficient initiation sites shared by all the cell lines analysed, which have a conserved DNA base composition (Akerman et al. 2020). On the other hand, low-efficiency initiation sites are believed to be those only used by a portion of the cell population, being stochastically activated in individual cells, and possibly serving as “dormant” origins (Jodkowska et al. 2022), the activation of which is prevented at least in part by SIRT1 deacetylase activity on TOPBP1 (Thakur et al. 2022). In the case of neighbouring fork stalling, dormant origins can activate and complete synthesis at un-replicated regions (Ge et al. 2007; Ibarra et al. 2008).

Although ORC, MCM and initiation site positions share common genetic and epigenetic features, a low overlap on their exact location has been noticed. In fact, analyses of ORC and MCM positions with respect to the initiation sites at core origins showed that ORC and MCM localise approximately at 500 bp and 200 bp upstream of the initiation site respectively, suggesting a physical separation of origin licensing and firing (Akerman et al. 2020). This could be explained due to the diffusive character of MCM DHs, or the fact that more than one MCM DH is probably loaded in at every ORC position, widening the region of the actual initiation (Hyrien 2016).

Opposite to the stochasticity of low-efficiency initiation sites, the broader replication units identified in the early vs late S phase by Repli-seq approaches indicate a tight and strict regulation of the replication program in time and space (Vouzas and Gilbert 2021). Initial analyses of Repli-seq data revealed a high correlation of early replication with highly transcribed, open chromatin regions corresponding to A-type genomic compartments, whereas transcriptionally silent, heterochromatic regions corresponding to B compartments are replicated later during the S phase (Ryba et al. 2010; Yaffe et al. 2010). Smaller replication domains inside early- or late-replicating regions can be observed and they strongly overlap with TADs (Pope et al. 2014). These analyses also identified the existence of “timing transition regions” (TTR), as those located at the boundaries between adjacent early and late-replication regions, which overlap with boundaries between A and B compartments (Ryba et al. 2010). Recent progress on Repli-seq approaches have greatly increased the resolution by analysing separately 16 different stages of the same S phase. These studies have led to the identification of narrow initiation zones (IZ; average of 200 kb) within early- and late-replicating domains (Zhao et al. 2020).

The consistent localisation of IZs observed by high-resolution Repli-seq in cell populations (Zhao et al. 2020; Klein et al. 2021; Emerson et al. 2022) and single cells (Dileep and Gilbert 2018) argues in favour of initiation factors being loaded and confined in specific genomic regions, while the exact sequence inside IZs where those factors are activated is likely stochastic in single cells, which might explain the variability found in replication origin data sets obtained by different methods. However, core and constitutive initiation sites (Akerman et al. 2020), initiation domains (Petryk et al. 2016) and narrow IZ (Emerson et al. 2022) have been consistently mapped at TAD boundaries. Given that TAD boundaries are sites of open chromatin due to their DNAse hypersensitivity and they are enriched in TSS (Pope et al. 2014; Akdemir et al. 2020), those could be sites of ORC and MCM-DH accumulation or assembly of proteins favouring initiation, and act as triggers to start replication of the whole TAD. Initiation of DNA synthesis at those positions might promote the firing of less efficient adjacent origins, which may be located in close proximity due to loop formation (Fig. 2). In this scenario, the role of cohesin in favouring MCM-DH accumulation and intra-TAD genome interactions might drive the domino-like effect observed at origins positioned nearby constitutive replication initiation sites (Löb et al. 2016).

A certain degree of variability in single cells is expected since TADs are dynamic entities mainly formed by loop extruding cohesin, not always placed at the same boundaries in all cells of a population (Gabriele et al. 2022). Nonetheless, placing highly efficient origins at TAD boundaries could constitute a safe mechanism to ensure replication of the whole TAD even when less efficient origins have problems to fire. Altogether, this might explain recent high-resolution Repli-seq data showing that cohesin inactivation specifically affects replication timing at strong-TAD boundaries containing CTCF in the early S phase, where constitutive replication origins tend to locate (Emerson et al. 2022). Moreover, a higher interconnection of initiation sites was shown to correlate with a higher efficiency of activation in mouse embryonic stem cells (Jodkowska et al. 2022). However, early initiation sites mapped by ORM show no firing interdependency with nearby origins inside the same DNA molecule, arguing against a role of origin physical proximity in promoting efficient, synchronised firing (Wang et al. 2021). Although some degree of stochasticity among highly efficient origins should probably be expected, technical limitations due to the experimental set-up—e.g. prolonged replication inhibition before release in S phase and origin mapping—may also account for some of the apparent discrepancies in the reported interdependency of origin firing.

Of note, this model does not imply that highly efficient initiation sites at TAD boundaries regulate the RT of that whole region, since deletion of a core origin such as MYC does not change the replication of the whole TAD, but rather only of the neighbouring region (Peycheva et al. 2022). Moreover, acute cohesin and CTCF depletions do not globally affect RT (Oldach and Nieduszynski 2019; Sima et al. 2019). Probably, the RT of a region is regulated by genetic features such as early replication control elements (ERCEs) (Sima et al. 2019) and the local epigenetic landscape, as shown upon acute depletion of RIF1 (Klein et al. 2021). ERCEs constitute short DNA sequences that simultaneously influence RT, transcription and genome architecture of a certain region. Given their enrichment in the open chromatin histone mark H3K27ac, it is plausible that these genetic elements affect replication timing through epigenetic regulation of those regions and increasing interaction hubs independently from cohesin and CTCF, thereby promoting the local confinement and engagement of initiation factors (Sima et al. 2019; Vouzas and Gilbert 2021).

Further insight in the importance of the epigenetic landscape for RT and vice versa was obtained by genetic ablation or inducible degradation of RIF1 (Klein et al. 2021). Initial biochemical work showed that RIF1 promotes the activity of the PPT1 phosphatase on MCM4, thereby counteracting DDK activity and limiting origin firing (Hiraga et al. 2017). Since RIF1 is mainly enriched in late-replicating regions (Foti et al. 2016), failure to limit origin firing in the absence of RIF1 would lead to their premature activation. Competition of late-replicating regions for replication initiation factors could explain the delayed firing of early replication origins and the apparent loss of early vs late RT in RIF1-defective cells. This deregulation of RT in RIF1 KO cells is associated with reduced genomic self-interactions and the overall loss of open and repressive histone marks in early and late-replicating regions, respectively. Of note, the subset of late-replicating regions maintaining their RT in the absence of RIF1 are further enriched in repressive histone marks and increase their genomic contacts, suggesting that their RT is controlled by a RIF1-independent mechanism. Importantly, genomic architecture, epigenomic landscape and RT are progressively lost after RIF1 inactivation, over multiple rounds of DNA replication (Klein et al. 2021), highlighting the importance of maintaining the RT for the transmission of epigenetic information to the replicated sister chromatids.

Besides genomic studies, super-resolution microscopy (SRM) of DNA synthesis events and replication factors has contributed to clarify how replication is regulated in the 3D genome in space and time. Combining single-molecule analyses of replication events by stretched DNA fibres and SRM of DNA synthesis proved possible to track individual replication events in intact nuclei by imaging approaches. Despite the physical proximity of origins inferred by genomic approaches, these studies showed that individual replication forks move untethered in the 3D genome (Chagin et al. 2016) and supported a model in which stochastic origin firing would lead to a domino-like effect on neighbouring origins to complete replication of each domain (Löb et al. 2016). Further work using higher resolution microscopy and sequential labelling of replication events showed that in the early S phase, initiation spreads from the base of replication domains to the periphery, while the opposite was true for later replicating domains (Su et al. 2020). Moreover, individual tracking of TADs together with origins, initiation events, and replication factors showed a chromatin re-arrangement of highly efficient origins from the TAD interior to the TAD boundary in the G1 to S transition (Li et al. 2021). This is not the case for low-efficiency origins. Surprisingly, the PCNA clamp was localised at TAD boundaries in G1 and re-localised as well during S phase. This process is mainly driven by chromatin-organising factors such as CTCF and cohesin, and the action of transcription shaping chromatin structure (Heinz et al. 2018; Li et al. 2021; Zhang et al. 2021). Altogether, these observations support a model in which highly efficient origins temporarily cluster to initiate DNA synthesis, but are physically separated once forks progress away from the origin.

Finally, once TADs are replicated, chromatin is reorganised to restore the gene expression pattern specific for the cell (Fig. 2). In a recent study, tracking of sister-chromatid specific interactions showed that at the G2 phase, intra-TAD interactions in *cis* (within the same molecule) are promptly re-established, while *trans* interaction between sister chromatids are mainly present at TAD boundaries (Mitter et al. 2020). This reorganisation is in line with the evidence that transcription is re-established approximately 2 h after replication of a specific region (Stewart-Morgan et al. 2019) (Fig. 2B, C). Moreover, data from chromatin conformation after RIF1 depletion in G1 and release at S phase shows that at late S phase, interactions at early-replicating domains are already affected in the absence of RIF1 (Klein et al. 2021). This data indirectly shows that early-replicating chromatin is actively reorganised after replication fork passage and before the S phase is finished (Fig. 2B, C).

## Stressed replication forks and global replication stress response

### Mechanisms of replication fork plasticity upon replication stress

Deregulation of origin firing or interference with progression or stability of ongoing replication forks leads to ssDNA accumulation and activation of the intra-S phase checkpoint, a condition commonly known as “replication stress” (RS). Cells react to RS by activating pathways to deal with the source of stress, to limit further DNA synthesis and prevent cell cycle progression in the presence of DNA damage. If the response to RS is defective or the source of stress exceeds available cellular resources, RS can lead to genome instability and cancer-associated rearrangements (Costa et al. 2022). Moreover, RS is at the heart of multiple cancer chemotherapeutic strategies, which aim to increase genomic instability in order to trigger the cellular apoptotic program and counteract tumour proliferation.

**Fig. 3:**
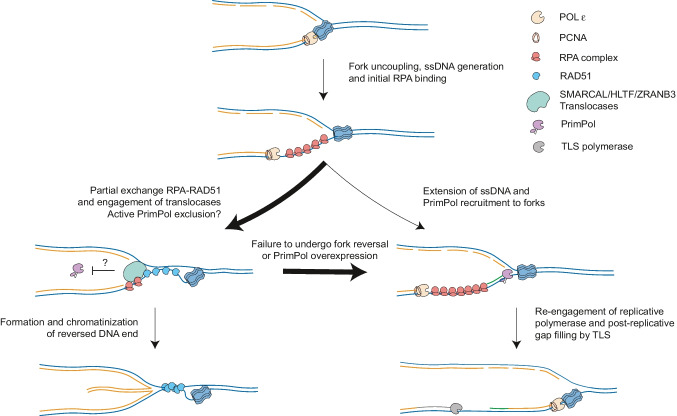
Pathways to deal with replication stress at replication forks. When forks are challenged, polymerase and helicase activities at the replisome are uncoupled, exposing ssDNA that is rapidly coated by RPA complex molecules. The most prominent way to deal with ssDNA at forks is to undergo fork reversal (left), by reannealing of parental DNA strands due to Rad51 activity, and annealing of newly synthesised DNA strands generating a reversed DNA end. Protection of the reversed DNA end to the action of nucleases ensures genomic stability is maintained under challenging conditions. Another mechanism to deal with replication stress is by the engagement and repriming activity of PrimPol at forks (right), which promotes primer generation at exposed ssDNA and re-initiation of DNA synthesis at a downstream position, favouring continuation of DNA synthesis and leaving small ssDNA gaps that need post-replicative filling by TLS. PrimPol-dependent repriming becomes prominent upon defective fork reversal or when PrimPol is overexpressed. One of the mechanisms dictating pathway choice at forks could be the extent and time of RPA at uncoupled forks, the ability of Rad51 to replace RPA, and the active exclusion of PrimPol from forks

Several studies in recent years have highlighted the surprising plasticity of replication fork architecture, as well as replisome composition, as key molecular determinants of the cellular response to RS (Berti et al. 2020). One of the earliest and global responses at replication forks to deal with impediments is replication fork reversal (RFR)—i.e. the conversion of replication forks into four-way junctions by coordinated unwinding and annealing events. By transiently pausing the replication fork, RFR actively limits DNA synthesis under unfavourable conditions, providing more time for template repair and an alternative template to continue DNA synthesis, thereby promoting DNA damage tolerance (Neelsen and Lopes 2015; Berti et al. 2020) (Fig. 3). Numerous factors—such as the SNF2-family DNA translocases (SMARCAL1, ZRANB3 and HLTF), the F-box DNA Helicase 1 (FBH1), and the central recombinase RAD51—mediate fork reversal and thereby active fork slowing in human cells (Bétous et al. 2012; Lemaçon et al. 2017; Mijic et al. 2017; Vujanovic et al. 2017; Bai et al. 2020; Liu et al. 2020). Intriguingly, the DNA end generated during reversal closely resembles a double-stranded break (DSB). This could explain that most factors involved in DSB repair are central players of RFR, and the fact that this DNA end can prime extensive nucleolytic degradation of nascent DNA in specific genetic backgrounds (Kolinjivadi et al. 2017; Lemaçon et al. 2017; Mijic et al. 2017; Taglialatela et al. 2017). The complexity of this process is highlighted by the fact that different pathways of fork protection co-exist and may have different pools of replication forks as targets. In essence, forks reversed by the action of SMARCAL1, HLTF and ZRANB3 are protected by BRCA2, FANCD2 and ABRO1, whereas BOD1L, VHL and other FA factors protect forks reversed by FBH1. Reversed forks can also be timely restarted by RECQ1-mediated reversed branch migration—linked to lesion removal or stress release via PARP1-mediated ADP-ribosylation (Berti et al. 2013; Zellweger et al. 2015)—or by a mechanism involving unwinding and controlled degradation of the reversed arm by the action of WRN helicase and the nuclease DNA2 (Thangavel et al. 2015).

Fork plasticity has been mostly studied in two different experimental settings: (a) under complete fork stalling (e.g. upon treatment with high concentrations of hydroxyurea, HU—a ribonucleotide reductase inhibitor which depletes the nucleotide pool—or Aphidicolin, Aph—a DNA polymerase alpha and delta inhibitor), or (b) under mild RS, using mild doses of genotoxic agents that allow a certain degree of DNA synthesis. Interestingly, the frequency of reversed forks found by single-molecule analysis of replication intermediates by electron microscopy is largely dose-independent (Chaudhuri et al. 2012; Zellweger et al. 2015). This implies that RFR is a highly transient state under mild RS in which synthesis, reversal and, at least in certain cases, nucleolytic degradation (Rainey et al. 2020) take place dynamically at replication forks, and likely extend to forks that are not directly challenged by DNA lesions or replication interference (Mutreja et al. 2018). In fact, under mild RS, the extent of fork progression reflects the relative contribution of synthesis, staling and degradation induced by that experimental condition (Vindigni and Lopes 2017; Berti et al. 2020).

Under mild RS sources that are in principle compatible with fork progression, such as UV or inter-strand crosslinks, the conserved primase activity of Primpol offers an alternative way to allow rapid replication restart. Repriming DNA synthesis counteracts fork reversal and promotes fast replication fork progression upon stress (García-Gómez et al. 2013; Mourón et al. 2013; Kobayashi et al. 2016; Guilliam and Doherty 2017; Bai et al. 2020; Piberger et al. 2020; Quinet et al. 2020; González-Acosta et al. 2021). However, Primpol-mediated repriming entails discontinuous DNA synthesis, leaving gaps behind replication forks, which require post-replicative gap-filling by translesion synthesis (TLS) polymerases (Quinet et al. 2021; Tirman et al. 2021) (Fig. 3). Different conditions impairing fork reversal lead to PrimPol-dependent unrestrained fork progression under mild RS. This is the case of RAD51- (Vallerga et al. 2015), SMARCAL1- (Quinet et al. 2020), ZRANB3- (Andrs et al. 2023) or HLTF-deficient cells (Bai et al. 2020). Furthermore, ATR was shown to promote global fork reversal upon different treatments (Mutreja et al. 2018), but can also shift the balance towards PrimPol-mediated repriming when cells are exposed to multiple doses of genotoxic treatments (Quinet et al. 2020). The choice between reversal and repriming was shown to impact the cellular sensitivity to genotoxic drugs, depending on the genetic background and DNA repair potential of the cells (Quinet et al. 2020). While PrimPol activity appears to contribute to drug resistance and proliferation when fork reversal is proficient (Quinet et al. 2021), detrimental accumulation of ssDNA gap has been observed when repriming prevails (e.g. upon PARP inhibition) or when gaps are not filled post-replicatively (e.g. upon BRCA-deficiency) (Cong and Cantor 2022). Overall, PrimPol-mediated repriming seems a favourable option for cells when prolonged fork stalling is likely to result in fork collapse and DNA damage accumulation. An alternative possibility to promote fork progression when fork reversal is not proficient is the engagement of “on the fly” TLS activity at replication forks (Guilliam and Yeeles 2020; Guilliam 2021). REV1-dependent unrestrained continuous DNA synthesis upon mild RS has been observed upon expression of the fork reversal-defective HLTF-HIRAN mutant (Bai et al. 2020) or upon the absence of the PARP1 interactor and activator CARM1 (Genois et al. 2021). Why certain conditions trigger PrimPol-dependent discontinuous synthesis or REV1-dependent continuous DNA synthesis remains to be elucidated.

### The emerging role of nuclear dynamics and 3D genome architecture in the RS response

Whether and how the RS response under complete fork stalling or synthesis-permissive mild RS conditions is organised or governed by genome architecture or factors involved in nuclear dynamics is largely unknown. To date, nuclear dynamic factors have been studied more extensively in yeast, in response to DSB generation or instability associated with the difficult-to-replicate ribosomal DNA (rDNA). Controlled homologous recombination at rDNA requires the re-localisation of the DSB to extranucleolar sites of repair (Torres-Rosell et al. 2007). This process depends on the post-translational modification of specific factors, such as Rad52 SUMOylation or phosphorylation and SUMOylation of the CLIP-cohibin complex, to release the DSB and prevent hyperrecombination at the rDNA locus (Torres-Rosell et al. 2007; Capella et al. 2021). Besides the release from the nucleolus, other studies reported that the relocation of unstable rDNA or telomeric DSBs to the nuclear periphery—via the association with components of the inner nuclear membrane or with the nuclear pores—promoted efficient repair and genome stability (Mekhail et al. 2008; Chung et al. 2015). Most repair events linked to re-localisation depend on different kinds of filaments within the nuclei or in the cytoplasm. For instance, DSB repair depends on the nuclear microtubule network and the action of kinesins in yeast (Chung et al. 2015; Oshidari et al. 2018) and on the formation of Rad51 filaments in humans (Cho et al. 2014). Moreover, a recent pre-print suggests that cytoplasmic filaments produce invaginations on the nuclear envelope, promoting its association with DSBs for efficient repair (Shokrollahi et al. 2023). Altogether, this evidence highlights the importance of subnuclear location and different filamentous structures to promote accurate and efficient DNA repair.

Recent evidence from higher eukaryotes reveals that a conserved active relocation of specific chromosomal loci within the nucleus, towards the nuclear periphery or even to reach direct contact with the nuclear pores, being important for the repair of DSBs or persistent obstacles to replication fork progression, often involve key components of nuclear architecture such as nuclear pores, nuclear actin filaments (F-Actin) and motor proteins (Bermejo et al. 2011; Horigome et al. 2014; Caridi et al. 2018, 2019; Kramarz et al. 2020; Lamm et al. 2020, 2021; Whalen et al. 2020). Importantly, clustering of distant DSBs-harbouring regions—even belonging to different chromosomes—was recently shown to generate a new genomic compartment (D compartment) and to stimulate DNA repair by promoting transcription of repair-related genes, at the expense of an increased risk of chromosomal translocations (Arnould et al. 2023).

Although these studies have focused on DNA breaks or prolonged fork stalling—frequently associated with fork collapse and breakage—recent findings suggest that also the immediate response to mild RS— which is compatible with residual fork progression and linked to fork plasticity—entails a coordinated response in the nuclei, likely involving nucleoskeleton components. As mentioned, RFR reaches saturating levels even at low doses of genotoxic treatments (Chaudhuri et al. 2012; Zellweger et al. 2015) and extends to forks that do not meet DNA lesions (Mutreja et al. 2018). This extension of the RS response from damaged/local to undamaged/global forks depends on a yet-elusive signalling mechanism driven by the central checkpoint kinase ATR (Mutreja et al. 2018). Although key effectors mediating this process are yet under investigation, factors involved in nuclear dynamics and genome folding are good candidates to drive RFR and to have an impact also in the RS response.

#### The cohesin complex

**Fig. 4:**
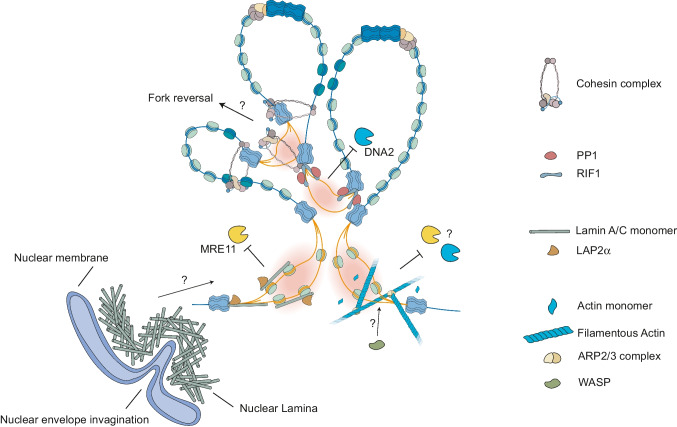
Possible roles of different genome organising and nuclear dynamic factors in the replication stress response. Cohesin recruitment at stressed replication forks could promote fork reversal, as it favours recombination-related processes. RIF1-PP1 complex is known to limit DNA2-driven fork degradation at stalled replication forks. Lamin A/C and Lap2α protect replication forks from MRE11 degradation. Possibly, the role of Lamin A/C in global fork protection is through monomeric Lamin A/C or the ability of the nuclear lamina to invaginate. Finally, nuclear F-Actin regulates the restart and stability of replication forks, and this functions dependent on the actin branching factors WASP and ARP2/3. The nucleases that actin-related proteins protect stalled replication forks from are still unknown. Whether WASP could also contribute to fork protection in an actin-independent manner through the regulation of RPA is still unknown

One of the candidates to mediate the RS response throughout the genome is the cohesin complex. As mentioned, cohesin regulates the DNA replication program, and different studies highlight that proper cohesin dynamics are crucial to maintain genome instability. For instance, the prevention of cohesin ring opening slows down replication forks and is rescued by reducing cohesin levels (Sakata et al. 2021). Moreover, depletion of the cohesin unloader WAPL or the associated factor PDS5 drives MRE11-dependent degradation of stalled replication forks due to deregulated cohesin accumulation at replication forks (Carvajal-Maldonado et al. 2019; Morales et al. 2020). Although deregulated levels of cohesin may impair fork progression, its rapid and controlled accumulation at replication forks upon HU-mediated fork stalling has been reported in different organisms (Tittel-Elmer et al. 2012; Dungrawala et al. 2015). In yeast, cohesin recruitment at forks is favoured by the MRX complex and chromatin remodellers that enhance chromatin accessibility at stalled replication forks (Tittel-Elmer et al. 2012; Delamarre et al. 2020). Furthermore, cohesin ubiquitylation and transfer behind replication forks seem required to promote fork stability (Frattini et al. 2017). The ability of cohesin to entrap the two sister chromatids and favour recombination-related processes (Losada 2014) could in principle favour fork reversal (Fig. 4). This is supported by the fact that SMARCAL1 depletion in a PDS5-deficient background restores replication fork progression rates (Morales et al. 2020). Whether this is indeed the case and whether this would depend on loop extrusion by cohesin or its interaction with the MCM complex are key open questions to investigate cohesin function at stressed replication forks. Hints emerge from recent studies on broken fork restart, which was shown to require WAPL-dependent release of cohesin at replication forks, after its NIBPL-dependent loading (Benedict et al. 2020) as proven in yeast (Delamarre et al. 2020). Moreover, cohesin increase at stalled forks was shown to be dependent on ATR activity (Dungrawala et al. 2015). Whether this possible function of cohesin depends on the reported ATM/ATR-dependent phosphorylation sites on SMC1 Ser957 and Ser966 previously involved in DNA damage response (Kim et al. 2002) remains to be addressed. Based on this indirect evidence, it seems likely that upon RS, and even upstream of prolonged fork stalling and breakage, cohesin might be targeted by ATR kinase and recruited to replication forks via loop extrusion, helping to rapidly coordinate the RS response within the whole replication domain. Once the stress is released, cohesin might need to be unloaded to allow proper fork restart.

#### RIF1

RIF1 has also been involved in both DSB repair by NHEJ though its interaction with 53BP1 (Blasiak et al. 2021) and protection of stalled replication forks in a 53BP1-independent manner, via its interaction with PPT1 (Garzón et al. 2019; Mukherjee et al. 2019). Upon HU treatment, RIF1 is modestly recruited to stalled replication forks likely thanks to its ability to bind cruciform-like structures such as the one present at a reversed fork. Importantly, RIF1 does not promote fork reversal, but rather protects reversed forks, preventing their DNA2-mediated unscheduled nucleolytic degradation, which is linked to genome instability and sensitivity to genotoxic treatments (Fig. 4) (Buonomo et al. 2009; Garzón et al. 2019; Mukherjee et al. 2019). It is likely that RIF1-associated phosphatase PP1 limits DNA2 hyperphosphorylation and activity at reversed forks (Mukherjee et al. 2019). Moreover, RIF1 was recently reported to protect replication forks in response to the DNA polymerase inhibitor Aph and proposed to do so by allowing efficient recruitment of cohesin to stalled forks (Lebdy et al. 2023). RIF1-mediated protection of stalled forks was also linked to its phosphorylation at consensus ATM/ATR SQ/TQ sites within its intrinsically disordered region (Balasubramanian et al. 2022).

#### Nuclear F-Actin and Lamin A/C

Among the novel players recently involved in the RS response are network proteins involved in nuclear dynamic processes, such as nuclear F-Actin or Lamin A/C. Growing evidence from DSB repair studies suggests that movement and/or relocation of DNA lesions in the nucleus plays a key role in DNA repair. In particular, DSB repair in active chromatin may require lesion clustering and coordination with transcription (Mitrentsi et al. 2020). Conversely, DSBs induced in repetitive, heterochromatic regions need relocation to the periphery of the heterochromatin domains and later to the nuclear envelope to allow for efficient repair, although control mechanisms differ in different organisms (Chiolo et al. 2011; Dion et al. 2012; Ryu et al. 2015; Horigome et al. 2016). Recent evidence has highlighted the contribution of actin-related proteins and nuclear actin polymerisation in DSB resection, relocation and clustering of DSBs, which determines the efficiency and mechanisms of the repair process (Caridi et al. 2018; Schrank et al. 2018; Marnef et al. 2019). Besides nuclear actin, also the nuclear lamina—a meshwork of filaments supporting the inner nuclear membrane, primarily composed of lamin A polymers—has been linked to DNA damage and repair. On the one hand, this thick filamentous structure protects condensed chromatin from mechanical insults thereby limiting DNA damage (Santos and Toseland 2021); at the same time, the nuclear lamina is strategically positioned to mediate nuclear membrane invaginations and functional interactions with nuclear pores, which reportedly assist a subset of DNA repair events (Marnef et al. 2019; Kramarz et al. 2020; Mitrentsi et al. 2020; Whalen et al. 2020; Wootton and Soutoglou 2021; Shokrollahi et al. 2023).

Recently, both nuclear F-Actin and lamin A/C were shown to assist replication fork stability upon prolonged fork stalling, avoiding excessive nucleolytic degradation or assisting repair of collapsed or broken forks (Singh et al. 2013; Graziano et al. 2018, 2021; Lamm et al. 2020). Upon prolonged HU and Aph treatments, stalled/broken replication forks were shown to associate with thick nuclear actin bundles and increase mobility/re-localisation due to the action of myosin II. This process is dependent on ATR kinase via mTORC1 activation, and actin-branching mediators such as WASP and ARP2/3 complex. Accordingly, the inability to polymerise nuclear actin impaired the ability of stalled replication forks to recover from damage and induced chromosomal instability (Fig. 4) (Lamm et al. 2020). In line with a role of actin-branching factors in the replication stress response, recent reports showed that WASP is an RPA-interacting factor that is recruited to stalled replication forks and, along with actin nucleation, modulates RPA availability, replication fork integrity and restart (Han et al. 2022). However, it is currently unclear whether the function of WASP upon fork stalling replication stress is possibly independent from actin, as recently described for the actin regulator Profilin-1 (PFN1) (Zhu et al. 2022) (Fig. 4). A role for nuclear myosin VI was also reported in the protection of stalled replication forks from DNA2-independent nucleolytic degradation, via recruitment of the fork protection factor WRNIP1 (Shi et al. 2023). Importantly, ARP2/3-dependent branched nuclear actin polymerisation was recently reported to orchestrate the immediate response to replication stress, mediating replication fork remodelling and thereby actively limiting fork progression upon mild replication interference, and thereby protecting genome stability (Palumbieri et al. 2023). Intriguingly, this novel function of nuclear F-actin is specifically needed to limit the access of Primpol to challenged forks, as all molecular phenotypes induced by defective nuclear actin polymerisation are rescued by Primpol inactivation (Palumbieri et al. 2023). Limiting access of distributive proteins was also proposed as key molecular function for the transient accumulation of heterochromatic marks that was recently reported at stalled forks (Gaggioli et al. 2023), supporting the concept that replication factories may need to be shielded from deregulated access, by specific modulation of chromatin compaction and local nuclear architecture. This will represent a novel, exciting avenue of research for future studies, that may also clarify crucial and clinically relevant cellular responses to chemotherapeutic treatments.

Along the same line, Lamin A/C was shown to be present at replication forks by isolation of proteins at nascent strand (iPOND) and further accumulated upon fork stalling. Prolonged depletion of Lamin A/C leads to unscheduled fork degradation upon complete fork stalling, due to the action of MRE11 nuclease (Fig. 4). In this context, Lamin A/C is required to deposit ssDNA binding proteins such as RPA and RAD51 at stalled forks (Graziano et al. 2021) and to promote their efficient restart (Singh et al. 2013; Graziano et al. 2021). Apart from being located in the nuclear periphery and its ability to form invaginations, some of the molecular functions of Lamin A/C rely on a minor, nucleoplasmic fraction of the protein, which appears to be regulated by specific post-translational modifications and co-factors. One of the key factors interacting with and regulating the nucleoplasmic pool of Lamin A/C is LAP2α (Naetar et al. 2017). Similarly to Lamin A/C, LAP2α is recruited to stalled replication forks and regulates RPA binding to damaged chromatin. Moreover, in the absence of LAP2α, stalled replication forks are unprotected and vulnerable to the nucleolytic action or Mre11 (Fig. 4) (Bao et al. 2022), hence phenocopying Lamin A/C depletion (Graziano et al. 2021). Surprisingly, the role of LAP2α in fork protection seems to be independent of its interaction with Lamin A/C, and rather depends on its direct interaction with RPA (Bao et al. 2022) (Fig. 4). Clarifying the relative contribution of nuclear lamina, formation of invaginations, nucleoplasmic Lamin A/C and LAP2α to the replication stress response will clearly require further investigation. In particular, for both Lamin A/C and nuclear F-Actin, it will be crucial to clarify whether their molecular role in the replication stress response entails the relocation of stalled/challenged forks, and whether it depends on well-characterised filamentous structures or novel, yet-elusive forms of filament polymerisation, as recently observed by live-cell super-resolution imaging for F-actin contribution to androgen signalling (Knerr et al. 2023). We consider most likely that genome architecture and nuclear dynamic factors influence locally the epigenetic landscape, regulating nucleosome remodelling and histone modifications, ultimately fine-tuning chromatin accessibility and ssDNA generation, and possibly mediating pathway choice at challenged replication forks, without necessarily implicating active transport or relocation of replicating DNA. Moreover, as discussed above, recent evidence suggests that specific components of the nuclear envelop may associate with sites DNA damage sites inside the nucleus, via specialised invaginations of the nuclear membrane (Shokrollahi et al. 2023). Intriguingly, lamin A/C was also recently implicated as critical phosphorylation target of ATR, mediating the rupture of nuclear and micronuclear membranes as a novel cellular response to accumulated DNA damage (Joo et al. 2023; Kovacs et al. 2023). These recent studies suggest that lamin A/C filaments participate in multiple ways to the cellular response to genotoxic stress and highlight a new fascinating avenue for further research.

One important limitation of most studies that have so far investigated the role of nuclear architectural components in the RS response is the use of experimental conditions that induce complete fork stalling conditions, while the role of these factors upon mild RS—arguably more likely to reflect partial replication interference by chemotherapeutic treatments—is still largely unknown. This mostly reflects the technical challenge that, differently from fork stalling conditions, the molecular responses are not extended and detectable at all replicating forks.

### Pathway choice at challenged replication forks

Investigating the contribution of actin, lamin, cohesin and other nuclear architectural components in RS conditions that are permissive for further fork progression will help clarify whether and how these factors specifically promote one or the other pathway of RS tolerance, such as RFR, repriming and/or restart.

Besides the recent evidence on nuclear F-Actin mentioned above, different points argue in favour of PrimPol being actively excluded. For instance, PrimPol overexpression in conditions of proficient fork reversal tilts the balance towards PrimPol-mediated repriming at challenged replication forks, suggesting that higher PrimPol availability outcompetes fork reversal-mediating factors (Quinet et al. 2020) (Fig. 3). Moreover, a TLS-independent function of Polymerase ι (iota) has been recently proposed as to prevent PrimPol action at stressed forks (Mansilla et al. 2023), although the molecular mechanism is still poorly understood. This might have clinical implications since PrimPol inhibition has been proposed as a possible target in cancer therapy in possible combination with chemotherapeutics. Hence, also understanding whether fork reversal and stability restores upon in PrimPol’s absence in a fork reversal-impaired background, or else those forks collapse, is crucial to completely understand this clinically relevant processes.

Regarding the determinant of pathway choice at stressed replication forks, the extent of RPA at forks has already been hypothesised to be a possible mediator (Quinet et al. 2021). High RPA/ssDNA ratio has been shown to inhibit repriming in vitro (Martínez-Jiménez et al. 2017). This would fit with the fact that, in vivo, upon immediate fork stalling, short ssDNA generation, fork reversal would be favoured over repriming. Short ssDNA between the replicative polymerase and helicase would justify the requirement of the recently identified CMG displacement function of RAD51 to favour translocase-mediated branch migration at forks (Liu et al. 2023), which is triggered by ssDNA-coating RPA (Berti et al. 2020). In the event of defective fork reversal, it is plausible that fork uncoupling is not limited and continues. This would extend the amount of ssDNA, which in this case could increase the amount of RPA and enhance PrimPol activity as shown in vitro (Martínez-Jiménez et al. 2017; Guilliam 2021) (Fig. 3). As mentioned before, those changes would be subtle under those conditions, so no major RPA accumulation would be observed. A promising approach to test this could be evaluating RPA—and its ATR-mediated phosphorylated form at Ser33—at replication forks by proximity ligation-related approaches (SIRF)(Roy et al. 2018), although PrimPol activity might mitigate RPA accumulation at forks by itself, reducing the increase expected in the absence of RFR.

Understanding what signal or mechanism drives RFR, repriming, or the use of TLS polymerases at challenged replication forks and evaluating how architectural and nuclear dynamic factors impact those processes could shed light on how cells respond to genotoxic treatments globally, having direct implication in cancer-relevant chemotherapy.

## Concluding remarks

Overall, while the evidence is consolidating on how genome organisation and nuclear architectural components regulate DNA replication initiation, it is becoming increasingly clear that nuclear dynamics is also a key determinant of the replication stress response. In analogy to earlier evidence in DNA repair, relocation of replicating chromatin upon prolonged fork stalling assists repair and restart of broken or collapsed forks and requires several well-known factors previously involved in nuclear architecture and support. More recent evidence involves genome organisation and nuclear dynamics also in the immediate response to mild replication interference. This may not involve stalling and collapse of affected forks, but rather active fork slowing and remodelling, rapidly extending throughout the nucleus. Moreover, these early responses likely entail local chromatin modulation at replication factories, rather than nuclear relocation of the affected genomic loci. Investigating these new fascinating mechanisms represents an important challenge for future studies and will require to exploit, combine and refine top-end imaging and genomic methods, in order to monitor rapid and transient responses that are likely to escape traditional investigation methods.

## Data Availability

Not applicable.
